# Natural IgG Anti-F (ab’)_2_ Autoantibody Activity in Children with Autism

**DOI:** 10.3390/biomedicines11030715

**Published:** 2023-02-27

**Authors:** Sylvie Tordjman, Annaëlle Charrier, Michel Kazatchkine, Pierre Roubertoux, Michel Botbol, Guillaume Bronsard, Stratis Avrameas

**Affiliations:** 1 Integrative Neuroscience and Cognition Center (INCC), 75006 Paris, France; 2 Pôle Hospitalo-Universitaire de Psychiatrie de l’Enfant et de l’Adolescent de Rennes (PHUPEA), Centre Hospitalier Guillaume Régnier (CHGR) et Université de Rennes 1, 35000 Rennes, France; 3 INSERM Unité 430, Immunopathologie Humaine, 75014 Paris, France; 4 Institut de Neurosciences Physiologiques et Cognitives, INPC.CNRS, 13256 Marseille, France; 5 School of Medicine, Université de Bretagne Occidentale (UBO), 29238 Brest, France; 6 Service Hospitalo-Universitaire de Psychiatrie de l’Enfant et de l’Adolescent de Brest, UBO et CHU de Brest, 29238 Brest, France; 7 Laboratory of Immunology, Pasteur Institute, 75015 Paris, France; 8 Hellenic Pasteur Institute, 11521 Athens, Greece

**Keywords:** autoimmune process, natural autoantibodies, IgG anti-F (ab’)_2_ autoantibodies, self-recognition, autism spectrum disorder

## Abstract

*Background:* Many and diverse autoimmune abnormalities have been reported in children with autism. Natural autoantibodies (NAAbs) play important immunoregulatory roles in recognition of the immune self. The objective of this study was to examine the presence of NAAbs in the sera of children with autism and across severity subgroups of autistic behavioral impairments. *Methods:* NAAbs were titrated in sera through an ELISA procedure in 60 low-functioning children with autism and 112 typically developing controls matched for age, sex and puberty. *Results:* Serum titers of IgG anti-F(ab’)_2_ autoantibodies were significantly lower in children with autism compared to typically developing controls (*p* < 0.0001), and were significantly negatively associated with autism severity (*p* = 0.0001). This data appears to be related more specifically to autism than to intellectual disability, given that IgG anti-F(ab’)_2_ levels were significantly negatively correlated with IQ scores in the autism group (*p* = 0.01). *Conclusions:* This is the first report in autism of abnormally low natural anti-F(ab’)_2_ autoantibody activity. The findings suggest a dysfunction of self-recognition mechanisms which may play a role in the pathogenesis of autism, especially for the severely affected children. These findings strengthen the hypothesis of an autoimmune process in autism and open the prospect of alternative medical treatment. Further neuroimmunological research is warranted to understand the exact mechanisms underlying this reduced natural IgG anti-F (ab’)_2_ autoantibody activity, and to assess its impact on the pathophysiology and behavioral expression of autism.

## 1. Introduction

Several immunological defects have been reported in autism spectrum disorder (ASD), a neurodevelopmental disorder involving social communication impairments and repetitive behaviors or interests in early childhood. These immunological defects include decreased complement proteins, a decreased number and altered functions of T cells (including regulatory T cells), an increased number and altered functions of natural killer (NK) cells, abnormal proliferative responses to mitogens, and inflammation in the gastrointestinal tract and post-mortem brain with activated microglia [1,2,3]. For example, the Schwartz et al. study on mice with a deficit in methyl-CpG binding protein 2 (*Mecp2*-null mice)—*Mecp2* is a gene found to be mutated in ASD and Rett syndrome—revealed an important role of phagocytosis by microglia in this animal model. Microglia from the *Mecp2*-null mice exhibit profoundly impaired phagocytic ability compared with wild-type microglia, suggesting that insufficient clearance of debris from the brain of these animals may contribute to developmental disorders; phagocytosis, the mechanism for clearance of both self and foreign cellular material, is critical to normal brain development and function, and an imbalance in this process of clearance and regeneration may contribute to several developmental abnormalities observed in ASD and Rett syndrome [1]. Additionally, abnormal numbers of monocytes or B cells were reported in children with ASD, but there are discrepancies in the results [1,4,5,6]. A review of studies on immune cell abnormalities in children with ASD is presented in Table 1. With regard to abnormalities in the gastrointestinal tract, De Theije et al. conducted a review on the importance of gastrointestinal problems in ASD, providing an overview of the possible gut-to-brain pathways with perspectives of pharmaceutical and/or nutritional approaches to therapy [3]. It is noteworthy that several studies [7] have reported altered intestinal microbiota composition in children with ASD compared to typically developing children (see Table 2 for a detailed review of studies on microbiota abnormalities in ASD). Interestingly, gut microbiota is known to have a crucial role in the development and functionality of innate and adaptive immune systems participating in organism homeostasis [8] (certain authors [9] define the overall function of the immune system as maintenance of homeostasis).

Furthermore, many studies have reported in ASD increased plasma and CSF levels of cytokines or serum concentrations of autoantibodies to caudate nucleus, cerebellar proteins or neurofilaments, myelin basic protein, neuron-axon filament protein, nerve growth factor, and α2 adrenergic receptors [3,12]. Recently, serum autoantibodies to other brain components, such as anti-nucleosome specific antibodies or cerebral folate receptor autoantibodies, have also been described in children with autism with possible therapeutic perspectives [13]. In addition, increased serum levels of anti-ganglioside M1 autoantibodies have been observed in children with autism [14], but the results are not totally congruent given that Moeller et al. [15] found no association between autism and anti-ganglioside M1 autoantibodies. Research on autoantibodies to serotonin (5-HT) receptors was of special interest in ASD given the well-replicated hyperserotonemia of autism [16], and that autoantibodies directed against human frontal cortical 5HT1a receptors were found in individuals with autism [17]. However, according to other studies, autoantibodies to 5-HT receptors can be found in the blood of children with autism as well as their typically developing controls, and therefore should not be considered characteristic of autism [16]. A review of studies on immune autoantibody abnormalities in ASD is presented in Table 3. Finally, clinical observations have shown a greater incidence of ear infections, fatal infections and allergic reactions in ASD children than in matched peers [18]. However, the generalizability of these results is hampered by small sample sizes of individuals with autism.

A possible immune dysfunction is of particular interest in ASD given the important role of the immune system in neurodevelopment such as synapse formation and neuronal plasticity [21], and the existence of alterations of synaptic communication and neuronal plasticity in autism [22]. We were particularly interested in the autoimmune hypothesis stating that autoimmune processes could affect the central nervous system and lead to mental disorders [18]. Thus, some studies identified children with pediatric autoimmune neuropsychiatric disorders (PANDAS) associated with streptococcal infections [23], including children with autism [24]. Interestingly, Perlmutter et al. [25] reported therapeutic benefits of intravenous immunoglobulins in 30 children with PANDAS, but this finding needs replication in larger trials, and longitudinal studies were not conclusive regarding the PANDAS concept [26]. A key role of the immune system is to recognize what belongs to the body, known as “self”, and what is foreign to the body, known as “non-self” [27]. Natural autoantibodies (NAAbs) have been designated as the antibodies present in the sera of healthy non-immunized individuals. NAAbs are produced by B cells and are likely to derive from proteins initially selected to build organisms that were adapted through evolution to recognize environmental constituents, while preserving their capacity to recognize self-antigens. This evolutionary process was intended to allow immune memory, immunoregulatory mechanisms and active homeostasis compatible with survival, the main goal of living organisms. Thus, NAAbs participate in the defense of organisms against infectious pathogens through effective recognition of environmental antigens, modulate the immune response and counteract tolerance breakdown and the development of autoimmune diseases through recognition of self-antigens, and also maintain tissue homeostasis. Studies performed during the last twenty years showed that most NAAbs are polyreactive, recognizing various self-molecules and participating in various physiopathological situations, showing either a beneficial or pathological role [28]. The main effects of NAAbs in the physiology and pathophysiology of the immune system are described in Table S1 (see Table S1 in the supplementary material available online; Table S1: Role of natural autoantibodies). Furthermore, the repertoire of NAAbs was found to be altered in several neurodegenerative diseases and mental disorders (such as schizophrenia or depressive disorder) [29].

During evolution, B-1 CD5^+^ cells acquired the ability to switch from polymeric IgM to monomeric IgG-type antibodies, thus allowing the production of polyreactive and mono-reactive IgG antibodies against either self- or non-self-antigens, mainly produced by B-2 cells [26]. The humoral innate immune response in higher vertebrates shares features of adaptive immunity in the requirement of an interaction between T and B cells. An important acquisition of the immune system function is the capacity to produce mono-reactive (antigen specific) IgG natural autoantibodies specific to either self-antigens, contributing to self-recognition (a major mechanism in immune regulation and immunological self-tolerance/autoimmunity) or non-self-antigens, contributing to recognition of environmental antigens (a major mechanism in the protection against infections). The active site and the idiotypic determinants of the antibodies are located on the IgG anti-F(ab’)_2_ fragments. Natural anti-F(ab’)_2_ autoantibodies play an important role in the recognition of the immune self [26]. We were particularly concerned by the possible role of NAAbs, including the natural anti-F(ab’)_2_ autoantibodies, in the alteration of self-recognition mechanisms in autism, given the high number and diversity of autoimmune abnormalities observed in children with ASD (see the very diverse autoimmune abnormalities described above and in Table 3).

In the present study, we hypothesized a dysfunction of the self-recognition properties of the immune system in autism. We examined the presence of NAAbs in the sera of children with autism compared to typically developing controls, and across severity subgroups of autistic impairments.

## 2. Materials and Methods

### 2.1. Participants

The study was conducted on 60 children with autism and 112 typically developing controls matched on age, sex and Tanner stage of puberty assessed by a pediatrician (Tanner stage 1: prepubertal; Tanner 2, 3, and 4: pubertal; Tanner 5: post-pubertal). Outpatients with autism, recruited from French day-care facilities, included 38 males and 22 females (mean age = 11.4 years, SD = 4.2; 26 prepubertal, 22 pubertal, 12 post-pubertal). The typically developing controls were recruited over a three-month period from a preventive medical center that they attended for a regular check-up. They were referred by the pediatrician working at the preventive medical center. The comparison group included 72 males and 40 females (mean age = 11.7 years, SD = 4.3; 47 prepubertal, 44 pubertal, 21 post-pubertal). The two groups did not differ significantly with respect to age, sex, and pubertal status.

Based on direct clinical observation of the child by two independent child psychiatrists (ST and a child psychiatrist of the French day-care centers), a diagnosis of autism was made according to the criteria of the DSM-5 (Diagnostic and Statistical Manual of Mental Disorders-5th edn, American Psychiatric Association), ICD-10 (International Classification of Diseases, World Health Organization) and CFTMEA (Classification Française des Troubles Mentaux de l’Enfant et de l’Adolescent), and was confirmed by the ratings of the autism diagnostic interview-revised (ADI-R) and the autism diagnostic observation schedule (ADOS) scales [30]. The ADI–R and the ADOS were administered and coded by two trained psychiatrists certified in the administration of these scales; they were the same two psychiatrists for the whole autism group, in order to homogenize the diagnostic approach. This approach, combining information from multiple sources based on clinical psychiatric judgment and the administration of the ADI–R completed by the ADOS, is recommended [31] and improves the confidence in the diagnosis of ASD [32].

All children with ASD and typically developing control children were sleeping in their parents’ house and were attending school for the control group, and day-care facilities for the autism group, on a daily basis from about 9am to 4pm. They were all Caucasian, had no history of encephalopathy or neuroendocrinological disease, and were determined to be physically healthy based on the examination by the pediatrician. Typically developing controls were determined to be free of any significant developmental, psychopathological or neurological disorder, and to have no family history of ASD. Similarly, no family antecedents of ASD or developmental disorder diagnosis were reported in the families of the autism group. However, of the 60 children with autism included in the study, 4 children had siblings with social communication impairments but who did not meet the full diagnostic criteria for ASD. All participants were unmedicated for at least 3 months before the blood drawing. The pediatric exam occurring the day of the blood drawing showed that all participants did not have any signs of inflammation or infection (especially no ear infections). In addition, a parental screening questionnaire was completed for the group with autism and the comparison group to rule out any history or allergy and infection occurring during the month before the blood drawing, as well as any family history of autoimmune disorders. The protocol was approved by the ethics committee of Bicêtre Hospital and written informed consent was obtained from parents.

### 2.2. Cognitive and Behavioral Assessments

The cognitive functioning of children with autism was assessed by two psychologists using the age-appropriate Wechsler intelligence scale and the Kaufman K-ABC. All children with autism were cognitively impaired (mean full scale IQ = 42.1, SD = 3.1, with a range of 40–58).

Diagnostic and behavioral assessments were performed using the autism diagnostic interview-revised (ADI-R) and the autism diagnostic observation schedule (ADOS) scales [30]. The ADI–R is an extensive semi-structured parental interview, and the ADOS is based on a direct observation of the child through a standardized semi-structured situation of games. The ADI–R and ADOS scales were used to assess major domains of autistic impairment: reciprocal social interactions, verbal and nonverbal communication, stereotyped behaviors and restricted interests. We used ADOS module 1, which is dedicated to individuals with limited or no speech.

Autism severity was assessed on the ADI-R scale completed by the ADOS scale, leading to an overall score of impairments for the combined social, communication and stereotypy domains (ranging from 1 to 3; knowing that the ‘0′ coding means “absence of autism”) using a methodology previously described [33]. An overall rating of autism severity, based on the social, behavioral and communication deficits, is also used with the childhood autism rating scale (CARS) [30]. The overall rating of autistic impairments for each patient was then used to dichotomize individuals according to autism severity. Individuals were grouped into mild-moderate (score 1–2) and severe (score 3) impairment. Inter-judge reliability with respect to the critical distinction between mild/moderate and severe impairment was excellent, with an inter-judge agreement of 95% observed between the two expert raters (given the large number of severely autistic children in our sample, it was possible to clearly distinguish the severely autistic subgroup).

### 2.3. Blood Drawing Procedures and Titration of NAAbs

Blood drawing for children with ASD (*n* = 60) occurred at the nearest general hospital rather than at the day-care center, so that the research procedure was not associated with the therapeutic milieu. Blood drawing for typically developing controls (*n* = 112) occurred at the preventative medical center. The blood drawing followed a standardized procedure to reduce possible stressful conditions. For all children with ASD and control children, parents were present during the blood drawing, and no white coats were worn in the presence of the children. Additionally, the children stayed in a playroom for 15 min before the blood drawing, and all phlebotomies were performed by the same nurse who was particularly experienced with handicapped children.

Blood was obtained by venipuncture (antecubital foci) performed between 8 and 9 a.m. The serum was separated by centrifugation (22 °C, 15 min, 4000 g) and frozen at −80 °C until assayed. NAAbs were titrated in sera by an ELISA procedure as previously described [26]. The intra- and inter-assay coefficients of variation were 5% and 10%, respectively.

### 2.4. Statistical Analysis

Group and autism severity subgroups comparisons of serum NAAbs titers were performed using analysis of variance (ANOVA) and two-tailed unequal variance t-tests. Correlations between NAAbs titers and age or IQ scores were calculated by Pearson correlation analyses.

## 3. Results

**Figure 1:**
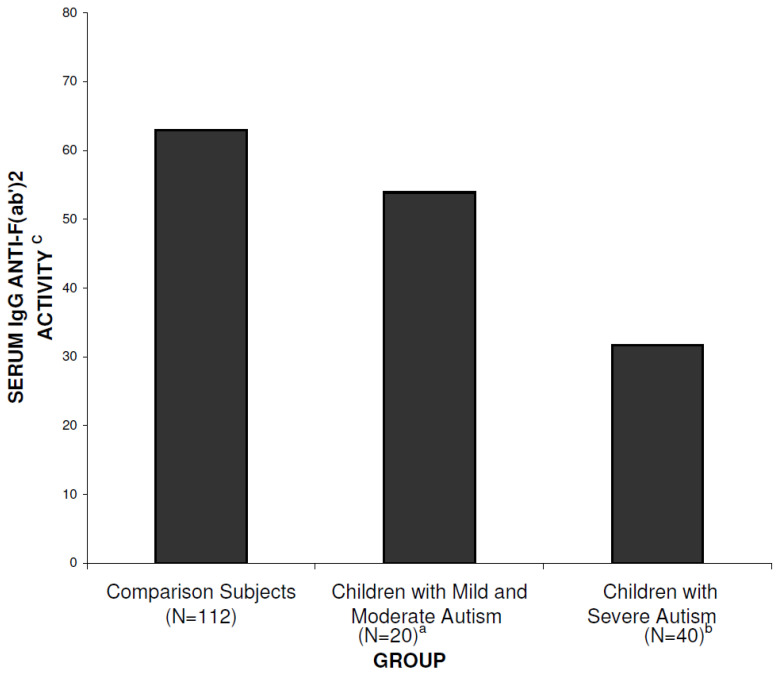
Mean serum IgG anti-F(ab’)_2_ titers in the autism group and the typically control group. Notes: ^a^ Significant difference between the mild and moderate autism group and the comparison group (t = 3.14, df = 129, *p* = 0.002). ^b^ Significant difference between the severe autism group and the comparison group (t = 7.53, df = 96, *p* < 0.0001) and between the severe autism group and the mild and moderate autism group (t = 6.53, df = 48, *p* < 0.0001). ^c^ Results are expressed as the percentage of the absorbance of the test serum compared with a reference pool of 800 normal sera.

The titers of autoantibodies to all the antigens of the panel tested did not differ between the autism group and the comparison group, and across severity subgroups of autism, except for the IgG anti-F (ab’)_2_ autoantibodies. Serum levels of IgG anti-F(ab’)_2_ fragments were significantly lower in children with autism (mean = 39.10, SD = 35.58, *n* = 60) than in comparison children (mean = 62.99, SD = 28.11, *n* = 112) (t = 4.50, df = 99, *p* < 0.0001). The ANOVA including sex, puberty and autism severity showed a significant relationship between IgG anti-F(ab’)_2_ levels and autism severity. Mean serum IgG anti-F(ab’)_2_ levels observed in individuals with “severe” autism (score of 3 on the overall rating) (mean = 31.70, SD = 20.20, *n* = 40) were lower than levels in individuals with “mild” to “moderate” autism (scores of 1 and 2) (mean = 53.90, SD = 5.2, *n* = 20) which were lower than in comparison subjects (62.99, SD = 28.11, *n* = 112), F = 10.38, df = 2, 154, *p* = 0.0001 (see Figure 1). There was no significant effect of gender, pubertal status or age on IgG anti-F(ab’)_2_ levels for either the autism or typically developing control group.

Finally, among individuals with autism, a negative and significant correlation of moderate size was observed between full scale IQ scores and IgG anti-F(ab’)_2_ levels (Pearson *r* = −0.37, *p* = 0.01). Therefore, there is a significant positive correlation between anti-F(ab’)_2_ autoantibody activity and severity of cognitive impairment in children with autism.

## 4. Discussion

The major finding of this study was that serum levels of IgG anti-F(ab’)_2_ autoantibodies were significantly lower in children with autism than in typically developing controls and were negatively associated with autism severity. These data cannot be explained by the cognitive impairments of children with autism, considering that IQ scores were significantly and negatively correlated with IgG anti-F(ab’)_2_ levels in the autism group. The significant negative correlation found in this study between anti-F(ab’)_2_ autoantibody activity and autism severity, taken together with the significant positive correlation between anti-F(ab’)_2_ autoantibody activity and severity of cognitive impairment, indicates that the findings are related more specifically to autism than to intellectual disability.

Our results are in line with Heuer et al.’s study [20] showing decreased levels of total IgG in 116 children with autism compared to 96 typically developing children and 32 non-autistic children with developmental delays, but they contradict Croonenberghs et al.’s report [19] of increased levels of total IgG, IgG2 and IgG4 in 18 individuals with autism compared to 22 typically developing controls. However, Croonenberghs et al. [19] conducted their study on small samples of older individuals (age: 13–19 years). In addition, these two prior studies did not specifically measure anti-F(ab’)_2_ autoantibodies. Interestingly, the decrease in total IgG levels observed in Heuer’s study was significantly associated, as in our study, with the global severity of autistic behaviors. Similarly, relationships between autism severity (social communication impairments and/or stereotypies) and immune dysfunction (abnormally higher levels of anti-ganglioside M1 autoantibodies, cytokines or chemokines, but also reduced levels of regulatory cytokines) were also reported in children with ASD [12,14,16].

Given that natural IgG anti-F(ab’)_2_ autoantibodies play a key role in self-recognition, our results suggest a markedly dysregulated autoreactivity in autism with a dysfunction in the recognition of the immune self and consequently in the ability to discriminate between self and non self. The hypothesis of a dysregulated autoreactivity in autism is also supported by Torrente et al., who have reported epithelial IgG deposition co-localizing with complement C1q in 23/25 children with regressive autism [34]. In addition, associations found between major histocompatibility complex (MHC) genes and autistic spectrum disorders are in favor of an autoimmune basis for autism. Indeed, the strongest associations detected within the MHC in ASD, for the null allele of complement C4B locus, the extended haplotype B44–S30–DR4 and the third hypervariable region of HLA–DR1, are known to be predisposed to the development of autoimmunity [34]. Furthermore, an abnormally high frequency of autoimmune disorders was found in family members of children with ASD [35]. Finally, our findings, taken together with these prior results, including the numerous reports of anti-brain autoantibodies observed in children with autism and previously described in the introduction, strengthen the hypothesis of an autoimmune process in autism.

The findings are consistent with the theory of links between the immune system and mental states [18]. In particular, our results suggesting an immune dysfunction of the self and non-self-recognition mechanisms in autism are of possible interest with regard to the autistic social communication impairments and the cognitive dysfunction of differentiation between self and non-self implied in the theory of mind in autism. In line with the hypothesis of ergodicity [36] that postulates the existence of similar mechanisms at different levels, relationships might exist between the immune network involving immune mechanisms of self and non-self-recognition and the neurocognitive network involving psychological mechanisms of self and non-self-recognition. Furthermore, Anspach and Varela [37] emphasized that the immune system involves properties of recognition, learning and memory related to a biological network sharing similarities with the cognitive network. The immune and nervous systems are both communication systems involving internal coordination and interactions in the context of an adaptable coherent unity [38].

Some study limitations should be acknowledged concerning the sample size of the autism group and the mechanisms underlying the immunological results. Indeed, the sample size of the autism group (*n* = 60) was smaller than the one of the typically developing control group (*n* = 120) due to the difficulty of the venipuncture in children with ASD reported by their parents during the preliminary information phase of the study; the blood drawing was a much easier situation for the control group as it was part of their regular check-up at the preventive medical center. Furthermore, the recruitment of children with ASD from day care facilities involves the participation of children with severe autistic impairments, given that French children with mild/moderate autism do not usually go to day care facilities on a daily regular basis. However, the participation of 60 children with ASD (including 40 children with severe autism and 20 children with mild/moderate autism) represents already a large total sample and quite large subgroups of children with ASD for biological studies in autism. The main limitations of the present study concern the exact mechanisms underlying these results which remain to be ascertained, analyzed and understood with regard to a more global dysfunction of the immune network, including reduced immune regulation through several mechanisms such as regulatory T cells’ activity (natural autoantibodies stimulate regulatory T cells known to inhibit autoimmune responses [39], and the number as well as function of regulatory T cells were also found to be abnormal in autism [10]) and gut microbiota composition (natural autoantibodies are highly dependent on the microbiota [40], and altered gut microbiota were found in autism [7]). Indeed, autoimmunity cannot be reduced to abnormal titers of specific idiotypes but is better understood as a dysfunction of the immune network [38]. However, it is noteworthy that natural anti-F(ab’)_2_ autoantibodies play important immunoregulatory roles in recognition of the immune self, and act therefore at a more general and underlying level than the numerous specific anti-brain autoantibodies observed in autism. Furthermore, the recombinatorial mechanisms in both the T cell and B cell receptors that have evolved to generate effective adaptive immunity require a coordinated system involving multiple complementary mechanisms to regulate and maintain unresponsiveness to self-antigens (immunological self-tolerance) [41]. It is probably necessary to re-configure the immune self through a more comprehensive integrative and dynamic approach, taking into consideration the complex immune interactions, the setting, and the full environmental context (the internal but also external environment) of immune recognition [42]. Along the same lines, based on a critical review of immunological studies in ASD, Gesundheit et al. proposed an integrative approach to immune and autoimmune mechanisms in ASD, combining brain antibodies, serum cytokines, family history, and immunogenetics [1].

Further neuro-immunological research on ASD, including the replication of reduced natural IgG anti-F(ab’)_2_ autoantibody activity and the study of relationships between natural autoantibody and gut microbiota composition or regulatory T cells’ activity, could lead to a better understanding of the mechanisms involved in the present immunological results and in the pathophysiology and pathogenesis of autism.

## 5. Conclusions

This is the first report in ASD of abnormally low natural anti-F(ab’)_2_ autoantibody activity significantly associated with autism severity. The findings strengthen the hypothesis of an autoimmune process in autism and suggest a dysfunction of self-recognition mechanisms which may play a role in the development of ASD, especially for severely affected children. Finally, these findings open the possibility of alternative medical treatment, at least for the most severely impaired individuals.

## Figures and Tables

**Table 1:** Studies of immune cell abnormalities in autism.

Studies	Measure	Individuals with Autism (*n*)	Controls (*n*)	Results
Warren et al., 1987 *	Investigation of the natural cytotoxic potential of peripheral blood mononuclear cells using K562 tumor cells as target cells	Children, adolescents, and adults with autism (age: 3 to 28) (*n* = 31)	Age-matched healthy volunteers (*n* = 15) Healthy adults (*n* = 23)	Reduced levels of cytotoxicity not correlated with quantitative alteration in NK cells.
Gupta et al., 1998 *	Th1-like (IL-2, IFN-γ) and Th2-like (IL-4, IL-6, and IL-10) cytokines examined in CD4^+^ and CD8^+^ T cells. Intracellular cytokines measured using specific antibodies to various cytokines and anti-CD4 or anti-CD8 monoclonal antibodies by FACScan	Children with autism (*n* = 20)	Typically developing (TD) children (*n* = 20)	Lower proportion of IFN-γ ^+^ CD4^+^ T cells, IL-2^+^CD4^+^ T cells (Th1), IFN-γ^+^CD8^+^ and IL-2^+^CD8^+^ T cells (TC1) in children with autism compared to TD controls. Increased IL-4^+^CD4^+^ T cells (Th2) and IL-4^+^CD8^+^ T cells (TC2) in autism. The proportion of IL-6^+^ CD4^+^, IL-6^+^CD8^+^, IL-10^+^CD4^+^, IL-10^+^CD8^+^ T cells was comparable in autism and control groups.
Sweeten et al., 2003 [4]	Leukocyte counts and plasma neopterin levels	Children with autism (*n* = 31)	TD children (*n* = 28)	Higher monocyte count and neopterin level in children with autism compared to TD children.
Saresalla et al., 2009 [1]	Immunophenotypic analysis; immunofluorescent staining and virological analysis	Children with autism (*n* = 20)	Non-autistic siblings (NAS) (*n* = 15). TD children (*n* = 20)	Decreased CD8^+^ T lymphocytes and diminution of CD8^+^ effector memory as well as CD4^+^ terminally differentiated lymphocytes in children with autism and NAS compared to TD children.
Enstrom et al., 2009 *	Gene expression screen and cellular functional analysis on peripheral blood	Children with autism spectrum disorder (ASD) (*n* = 52)	TD children (*n* = 27)	Upregulated RNA expression of NK cell receptors and effector molecules in ASD. Flow cytometric analysis of NK cells demonstrated increased production of perforin, granzyme B, and interferon gamma (IFNγ) under resting conditions in ASD children. Reduced cytotoxicity of NK cells and lower presence of perforin, granzyme B, IFNγ in NK cells in ASD children compared to TD controls.
Morgan et al., 2010 [2]	Microglial cell density stereologically estimated via optical fractionator and average somal volume quantified via isotropic nucleator in the dorsolateral prefrontal cortex	Children, adolescents, and adults with autism (age: 3 to 41) (*n* = 13)	TD children, adolescents, and adults (age: 1 to 44) (*n* = 9)	Microglia appeared activated in 9 out of 13 individuals with autism. Morphological alterations included somal enlargement, process retraction and thickening, and extension of filopodia from processes. Average microglial somal volume was significantly increased in white matter, with a trend in gray matter. Microglial cell density was increased in gray matter.
Ashwood et al., 2011 [6]	Number and phenotype of circulating blood cells (644 primary and secondary variables, including cell counts and the abundance of cell surface antigens) assessed using microvolume laser scanning cytometry	Children with autism (*n* = 70), including low functioning children (*n* = 35) and high functioning children (*n* = 35)	TD children (*n* = 35)	The absolute number of B cells per volume of blood was over 20% higher for children with autism, and the absolute number of NK cells was about 40% higher. The absolute number of T cells was not different across groups, but the number of cellular activation markers, including HLA-DR and CD26 on T cells and CD38 on B cells, was significantly higher in the autism group compared to the control group.
Ashwood et al., 2010 [10]	Cellular responses following in vitro stimulation of peripheral blood mononuclear cells with PHA and tetanus	Children with ASD (*n* = 66)	TD children (*n* = 73)	Increased production of GM-CSF, TNFα, IL-13 and decreased production of IL-12p40 following PHA stimulation in the ASD group compared to the TD control group. Increased pro-inflammatory or TH1 cytokines associated with greater impairments in ASD core features and aberrant behaviors; production of GM-CSF and TH2 cytokines was associated with better cognitive and adaptive function. Reduced frequency in ASD of CD3+, CD4+ and CD8+ T cells expressing activation markers CD134 and CD25 (but not CD69, HLA-DR or CD137) following stimulation.
Torres et al., 2012 *	Killer-cell immunoglobulin-like receptor (KIR) proteins expressed on NK cells using the whole genome amplification and HLA genotyping	Children with autism (*n* = 158)	TD children (*n* = 176)	Increased expression in four activating KIR genes (2DS5, 3DS1, 2DS1 and 2DS4) and the cognate HLA-C2 ligand of the activating KIR gene 2DS1.

* Cited in Mead and Ashwood (2015) [3].

**Table 2:** Studies of microbiota abnormalities in autism or related animal models.

Studies	Measure	Individuals with Autism or Animal Models (*n*)	Controls (*n*)	Results
Sandler et al., 1999 ^a^	An intervention trial using a minimally absorbed oral antibiotic. Short-term improvement was noted using multiple pre- and post-therapy evaluations: Behavior and communication analog rating scales Quantitative fecal flora stool specimen data from children with autism prior to vancomycin therapy	Children with regressive-onset autism (*n* = 11) Children with autism (*n* = 4)	Healthy adults (*n* = 104)	An open-label vancomycin trial indicates the possibility of a gut flora-brain connection in a subset of children with autism and diarrhea. Improvement for children with autism in communication and behavior following the vancomycin trial. Anaerobic cocci were absent from the stools of each of the 4 children with autism tested whereas they were present in 93% of the adults’ specimens.
Finegold et al., 2002 ^a^	Comparison of faecal flora; clostridial counts	Children with regressive autism (*n* = 13)	Typically developing (TD) children (*n* = 8)	Clostridial counts in fecal flora were higher in children with regressive autism; children with autism had 9 species of *Clostridium* not found in TD children, whereas controls yielded only 3 species not found in children with autism.
Parracho et al., 2005 ^b^	Fecal bacterial populations were assessed using a culture-independent technique, fluorescence in situ hybridization, with oligonucleotide probes targeting predominant components of the gut flora	Children with autism spectrum disorder (ASD) (*n* = 58)	Healthy siblings (*n* = 12) TD children (*n* = 10)	The fecal flora of ASD patients contained a higher incidence of the *Clostridium histolyticum* group of bacteria compared to healthy children. The non-autistic sibling group had an intermediate level of the *Clostridium histolyticum* group, which was not significantly different from either of the other subject groups.
Finegold et al., 2010 ^b^	Fecal microbial flora using the bacterial tag encoded FLX amplicon pyrosequencing (bTEFAP) procedure	Children with autism and gastrointestinal symptoms (*n* = 33)	Healthy siblings (*n* = 7) TD children (*n* = 8)	High levels of *Bacteroidetes* and *Desulfovibrio* species were found in the severely autistic group.
MacFabe et al., 2010 ^a^	Effects of propionic acid (PPA) on brain tissue and behavior (social, restricted interest and reversal in the T-maze task)	Rats in propionic acid (PPA) (*n* = 20)	Rats in phosphate-buffered saline (PBS) (*n* = 17)	PPA rats showed reactive astrogliosis and activated microglia (indicating an innate neuroinflammatory response) and impairment behavior (restricted behavioral interest to a specific object among a group of objects, impaired social behavior, and impaired reversal in a T-maze task) compared to PBS controls.
Adams et al., 2011 ^b^	Assessment of gastrointestinal flora, gastrointestinal status and yeast using standard culture growth-based techniques and other markers of digestive function	Children with ASD (*n* = 58)	TD children (*n* = 39)	Abnormally low levels of lysozyme and total short chain fatty acids in the ASD group, including acetate, proprionate, and valerate. The ASD group had lower levels of species of *Bifidobacter* and higher levels of species of Lactobacillus, but similar levels of other bacteria and yeast compared to the control group.
Williams et al., 2011 ^b^	Expression of human genes involved in carbohydrate digestion and transport along with bacterial community composition in intestinal biopsies. Composition of intestinal bacteria was examined using metagenomic analysis	Children with autistic disorder (AD) and gastrointestinal disease (*n* = 15)	TD children with gastrointestinal disease (*n* = 7)	Deficient ileal transcripts encoding disaccharidases and hexose transporters in the AD group, and impaired primary pathway for carbohydrate digestion and transport in enterocytes associated with expression of the intestinal transcription factor, CDX2. Composition of dysbiosis was decreased in *Bacteroidetes* but increased in the ratio of *Firmicutes* to *Bacteroidetes* and in *Betaproteobacteria*. Expression levels of disaccharidases and transporters associated with the abundance of affected bacterial phylotypes.
Williams et al., 2012 ^b^	Novel PCR-based methods for detection, quantitation, and phylogenetic characterization of *Sutterella* species in intestinal biopsy samples	Children with autism and gastrointestinal dysfunction (*n* = 23)	TD children with gastrointestinal dysfunction (*n* = 9)	*Sutterella* 16S rRNA gene sequences were found in 12 of 23 children with autism but in none of the controls. High levels of intestinal and mucoepithelial-associated *Sutterella* species were associated with gastrointestinal disturbances in children with autism.
Wang et al., 2012 ^a^	Concentrations of short chain fatty acids, phenols and ammonia measured in fecal samples	Children with ASD (*n* = 23)	TD children (*n* = 31)	Levels of fecal total short chain fatty acid, fecal ammonia, fecal acetic, butyric, isobutyric, valeric and isovaleric acids were higher in the ASD group compared to the control group. Similar levels of fecal phenol and pH between groups.
Thomas et al., 2012 ^a^	Effects of treatments (intracerebroventricular infusion procedure) on behavior and phospholipid components: automated activity monitors, behavioral testing, lipid extraction, electrospray ionization mass spectrometry analysis	Long-Evans rats with propionic acid (PPA) treatment (*n* = 12)	Long-Evans rats with phosphate-buffered saline (PBS) (*n* = 12)	PPA-rats displayed abnormal ASD-like behaviors associated with alteration in brain and blood phospholipid molecular species.
Gondalia et al., 2012 ^b^	Parental questionnaires on child diagnosis and symptoms, including gastrointestinal symptoms. Collection of stool samples for analyses: DNA extraction, bacterial identification (numbers of species, phylum levels, etc.)	Children with autism but without gastrointestinal dysfunction (*n* = 23) Children with autism and gastrointestinal dysfunction (*n* = 28)	TD siblings (*n* = 53)	*Firmicutes* (70%), *Bacteroidetes* (20%) and *Proteobacteria* (4%) were the most dominant phyla in samples.
Kang et al., 2013 ^b^	Pyrosequencing the V2/V3 regions in bacterial 16S rDNA from fecal DNA samples	Children with autism and gastrointestinal disorders (*n* = 20)	TD children without gastrointestinal disorders (*n* = 20)	The presence of autistic symptoms, rather than the severity of gastrointestinal symptoms, was associated with less diverse gut microbiomes. There were significantly lower abundances of *Prevotella*, *Coprococcus* and unclassified *Veillonellaceae* in children with autism.
Hsiao et al., 2013 [11]	Microbial composition Gut permeability ASD-related defects in communicative, stereotypic, anxiety-like and sensorimotor behaviors	Offspring from MIA (maternal immune activation) pregnant C57BL/6N mice treated with human commensal *Bacteroides fragilis*	Offspring from MIA pregnant C57BL/6N mice treated with vehicle in 1.5% sodium bicarbonate	Oral treatment of MIA offspring with *Bacteroides fragilis* corrects gut permeability, alters microbial composition and improves ASD-related defects in communication, stereotyped, anxiety-like and sensorimotor behaviors.

^a^ Cited in Cryan and Dinan (2012) [7]; ^b^ Cited in Hsiao et al. (2013) [11].

**Table 3:** Studies of immune autoantibody abnormalities in autism.

Studies	Measure	Individuals with Autism (*n*)	Controls (*n*)	Results
Croonenberghs et al., 2002 [19]	Total serum protein (TSP) and serum concentrations of albumin, alpha1 globulin, alpha2 globulin, beta globulin and gamma globulins, IgA, IgM and IgG and the IgG subclasses IgG1, IgG2, IgG3 and IgG4	Adolescents with autism (age: 13 to 19) (*n* = 18)	Typically developing (TD) adolescents (*n* = 22)	Increased concentrations of TSP in autism related to increased serum concentrations of albumin; significant correlations between gamma globulin and social problems, especially social withdrawal. Increased serum concentrations of IgG, IgG2 and IgG4 and albumin were significantly correlated with social problems and social withdrawal, respectively.
Croonenberghs et al., 2002 *	Production of interleukin (IL)-6, IL-10, the IL-1 receptor antagonist (IL-1 RA), interferon (IFN)-γ and tumor necrosis factor (TNF)-α by whole blood and serum concentrations of IL-6, IL-2 receptor (IL-2R) and IL-1RA	Adolescents with autism (age: 12 to 18) (*n* = 13)	TD adolescents (*n* = 13)	Increased production of IFN-γ, IL-1RA, IL-6 and TNF-α in adolescents with autism. Similar serum concentrations of IL-6, IL-2R and IL-1RA between adolescents with autism and TD adolescents.
Cabanlit et al., 2007 *	IgG antibodies against protein extracts from specific regions of the human adult brain, including the hypothalamus and thalamus	Children with autism spectrum disorder (ASD) (*n* = 63)	TD children (*n* = 63) Healthy siblings (*n* = 25) Children with other developmental disabilities (*n* = 21)	Multiple brain-specific autoantibodies are present at significantly higher reactivity in ASD children.
Heuer et al., 2008 [20]	Plasma levels of immunoglobulin (IgG, IgM, IgA and IgE)	Children with autism (*n* = 116)	TD children (*n* = 96) Children with developmental delays (DD) children (*n* = 32) Children with ASD but not full autism (*n* = 27)	Reduced level of IgG in children with autism compared to TD and DD children. Reduced level of IgM in children with autism compared to TD children. IgG and IgM levels were negatively correlated with aberrant behavior checklist scores for all children.
Enstrom et al., 2009 *	Circulating plasma levels of IgG1, IgG2, IgG3 and IgG4	Children with autism (*n* = 114)	TD children (*n* = 96) DD children (*n* = 31)	Increased levels of the IgG4 subclass in children with autism compared to TD children and DD children
Li et al., 2009 *	Levels of cytokines in frozen human brain tissue	Children, adolescents and adults with ASD (age: 4 to 37) (*n* = 8)	TD children, adolescents, and adults (age: 4 to 39) (*n* = 8)	Increased brain levels of proinflammatory cytokines (TNF-α, IL-6 and GM-CSF), Th1 cytokine (IFN-γ) and chemokine (IL-8) in individuals with ASD compared to TD controls. No significant difference for levels of Th2 cytokines (IL-4, IL-5 and IL-10) in the two groups. Increase in the Th1/Th2 ratio in individuals with ASD.
Saresalla et al., 2009 [1]	Intracellular cytokines, perforin and granzyme production; monoclonal antibodies, autoantibodies, and virological analysis	Children with autism (*n* = 20)	Non-autistic siblings (NAS) (*n* = 15) TD children (*n* = 20)	Increased proinflammatory and interleukin-10-producing immune cells in children with autism and NAS compared to TD children. Detection of serum autoantibodies (GM1) in 10% of children with autism.
Onore et al., 2009 *	Cellular release of IL-17 and IL-23 following an in vitro immunological challenge of peripheral blood mononuclear cells (PBMC)	Children with ASD (*n* = 34)	TD children (*n* = 26)	Following stimulation, the concentration of IL-23, but not IL-17, was significantly lower in the ASD group compared to the TD group.
Enstrom et al., 2010 [5]	Stimulated cell cultures in vitro of peripheral blood monocytes with distinct Toll-like receptors (TLR) ligands: TLR 2 (lipoteichoic acid; LTA), TLR 3 (poly I:C), TLR 4 (lipopolysaccharide; LPS), TLR 5 (flagellin), and TLR 9 (CpG-B). Pro-inflammatory cytokine responses for IL-1b, IL-6, IL-8, TNFα, MCP-1, and GM-CSF were determined by multiplex Luminex analysis.	Children with ASD (*n* = 17)	TD children (*n* = 16)	Increased pro-inflammatory IL-1b, IL-6, and TNFα responses following TLR 2 stimulation, and IL-1b response following TLR 4 stimulation in children with ASD compared to TD children. Decreased IL-1b, IL-6, GM-CSF, and TNFα responses following TLR 9 stimulation in children with ASD compared to TD children.
Ashwood et al., 2011 [12]	Assessment plasma cytokine production by multiplex Luminex analysis	Children with ASD (*n* = 97)	TD children (*n* = 87) Children with developmental disabilities other than autism (*n* = 39)	Increased levels of cytokines (IL-1b, IL-6, IL-8 and IL-12p40) in the ASD group, especially for the regressive form of ASD, compared to the TD group.
Wei et al., 2011 *	An in vitro adenoviral gene delivery approach was used to over-express IL-6 in cultured cerebellar granule cells. Cell adhesion and migration assays, DiI labeling, TO-PRO-3 staining and immunofluorescence were used to examine cell adhesion and migration, dendritic spine morphology, cell apoptosis and synaptic protein expression, respectively.	Children with autism (*n* = 6)	TD children (*n* = 6)	IL-6 was increased in the cerebellum of children with autism. IL-6 over-expression in granule cells caused impairments in granule cell adhesion and migration, and stimulated the formation of granule cell excitatory synapses (without affecting inhibitory synapses), but had little effect on the formation of dendritic spines or granule cell apoptosis.
Goines et al., 2011 *	Measured in banked serum of levels of 17 cytokines and chemokines using Luminex technology	Women at 15 to 19 weeks of gestation who gave birth to a child with ASD (*n* = 84)	Women at 15 to 19 weeks of gestation who gave birth to a DD child (*n* = 49) Women at 15 to 19 weeks of gestation who gave birth to a TD child (*n* = 159)	Elevated concentrations of IFN-γ, IL-4 and IL-5 in mid-gestation maternal serum were significantly associated with a 50% increased risk of ASD. Elevated concentrations of IL-2, IL-4 and IL-6 were significantly associated with an increased risk of DD without autism.
Al-Ayadhi et al., 2012 *	Serum levels of IL-17A by ELISA	Children with autism (*n* = 45)	TD children (*n* = 40)	Abnormally higher serum IL-17A levels in children with autism associated with autism severity.
Ricci et al., 2013 *	Serum levels of cytokines and BDNF	Children, adolescents and young adults with ASD (age: 2 to 21) (*n* = 29)	TD children, adolescents, and young adults (*n* = 29)	Elevated levels in the ASD group of pro-inflammatory cytokine (including interleukin-1, interleukin-6, interleukin-12, interleukin-23, tumor necrosis factor-α and BDNF).

^*^ Cited in Mead and Ashwood (2015) [3].

## Data Availability

Data sharing not applicable.

## Supplementary Materials

The following supporting information can be downloaded at: https://www.mdpi.com/article/10.3390/biomedicines11030715/s1, Table S1: Role of natural autoantibodies.
