# Supplementary material for: Natural IgG Anti-F (ab’)2 Autoantibody Activity in Children with Autism
Source: Biomedicines. 2023 Feb 27;11(3):715. doi: 10.3390/biomedicines11030715 (PMC10045297; doi:10.3390/biomedicines11030715)
Supplement: Supplementary file 1 [file biomedicines-11-00715-s001.zip › biomedicines-2141957-supplementary.pdf]

Table S1. Role of Natural Autoantibodies.

**Physiology**	**Pathology (beneficial and detrimental interference in case of uncontrolled response)**
Host defense<br>• Bacteria<br>• Viruses<br>• Other pathogenic agents	Inflammation, infection (chronic infection and sepsis), and autoimmune diseases
Maintenance of Tissue Homeostasis<br>• Clearance of catabolic products, apoptotic and necrotic cells, as well as senescent cells<br>• Anti-tumor activity (tumor-cell clearance)<br>• Modulation of cell functions: catalytic, hormonal, nucleic acids and protein synthesis	Cancer
Immunomodulation<br>• Cytokines<br>• Dendritic, B and T cells<br>• Presentation of antigens to T cells<br>• Granulocytes<br>• Bridge innate and adaptive immune responses	Cardiovascular diseases<br>• detrimental IgG anti-phosphorylcholine antibodies and protective IgM<br><br>Neurodegenerative diseases<br>• Remyelinization and neurite extension<br>• Recognition of beta-amyloid degradation products<br>• Recognition of beta-integrins<br><br>Tissue transplantation
